# Comparative Analysis of Animal Welfare in Three Broiler Slaughterhouses and Associated Farms with Unsatisfactory Slaughterhouse Results

**DOI:** 10.3390/ani14172468

**Published:** 2024-08-25

**Authors:** Sónia Saraiva, Sara Santos, Juan García-Díez, João Simões, Cristina Saraiva

**Affiliations:** 1 Animal and Veterinary Science Center (CECAV), University of Trás-os-Montes e Alto Douro (UTAD), Quinta de Prados, 5000-801 Vila Real, Portugal; juangarciadiez@gmail.com (J.G.-D.);; 2 Department of Veterinary Sciences, School of Agricultural and Veterinary Sciences, University of Trás-os-Montes e Alto Douro (UTAD), 5000-801 Vila Real, Portugal; 3 Associate Laboratory for Animal and Veterinary Science (AL4AnimalS), 1300-477 Lisboa, Portugal

**Keywords:** ascites, breast burn, broiler flock, cellulitis, emaciation, farm, footpad dermatitis, welfare, hock burn, slaughter

## Abstract

**Simple Summary:**

Data collected during meat inspection in slaughterhouses are invaluable for monitoring and surveilling the welfare and health of broilers These inspections help to ensure that meat products meet regulatory standards for food safety and animal welfare. The objective of this study was to assess, in three different slaughterhouses, the health and welfare of different commercial broiler flocks based on welfare indicators observed from the animals. Emaciation, dirty feathers (DFs), footpad dermatitis (FPD), hock burn (HB), breast burn (BB), breast blister, breast ulcer, ascites, septicemia/abnormal color, cellulitis, extensive traumatisms (affecting at least two distinct body parts), dead on arrival (DoA) and condemnation rate were the indicators measured. The results were considered unsatisfactory if thresholds were exceeded for DoA (>0.5%), condemnation rate (>4%), or extensive traumatisms (>2%) or grades 1 or 2 were achieved for DFs, FPD, HB, BB, breast blister, and breast ulcer. Eleven houses exceeded the thresholds for at least two welfare indicators, leading to audits of those farms. During farm audits, the main weaknesses identified included environmental conditions such as house temperature, ventilation, lighting programs, high stocking densities, structural infiltrations, water leakage and litter quality.

**Abstract:**

The objective of this study was to assess the health and welfare of 70 commercial broiler flocks (transport batches) in three distinct slaughterhouses based on various indicators including emaciation, dirty feathers (DFs), footpad dermatitis (FPD), hock burn (HB), breast burn (BB), breast blister, breast ulcer, ascites, septicemia/abnormal color, cellulitis, extensive traumatisms, dead on arrival (DoA) and condemnation rate. Assessment scales ranging from 0 (absence) to 2 (severe) were used for DFs, FPD, and HB, while a 0 (absence) to 1 (present) scale was applied to BB, breast blisters, and breast ulcers. The prevalence of total condemnation causes (emaciation, ascites, septicemia/abnormal color, cellulitis, and extensive traumatism) and DoA were recorded and presented in percentages. Three flocks presented condemnation rates higher than 4% and 11 flocks presented DoA rates higher than 0.5%. Twenty-one flocks achieved grade 1 (warning) for FPD and 14 achieved grade 2 for FPD (alarm). Extensive trauma was observed in 0.01% of the slaughtered animals, and no flock reached the threshold of 2%. Breast blisters and breast ulcers lesions were not observed in the studied flocks. The significant positive associations observed for the presence of severe footpad dermatitis (FPD2), severe hock burns (HB2), and breast burns (BB1) indicate simultaneous occurrences. Absences of hock burns (HB0) and breast burns (BB0) were also associated. Eleven houses that obtained the worst results for welfare indicators at slaughterhouses were audited. FPD, HB, DoA, and the condemnation rate were the most crucial indicators for identifying farms with inadequate welfare conditions. These indicators should be systematically integrated into the welfare monitoring of broilers in slaughterhouses. Audits conducted on farms detected some noncompliance with regulatory welfare standards and suggested improvements in environmental and structural conditions, as well as the reduction in stocking densities and improvements in the water systems.

## 1. Introduction

The Council Directive 2007/43/EC established minimum rules for the protection of broilers. From 1 July 2010, official veterinarians monitor the welfare conditions of broilers in all batches for slaughter, using indicators such as “abnormal levels of contact dermatitis, parasitism or systemic illness” to identify farms most likely to have poor welfare [1,2]. Contact dermatitis is frequent in intensive chicken production, affecting skin areas of the pads, joints, and breast due to skin contact with unsuitable or irritating litter materials [3,4]. Also referred to as “ammonia burns”, footpad dermatitis (FPD), hock burns (HB), and breast burns (BB) are considered welfare indicators of broiler housing conditions resulting in pain and discomfort to the bird [5]. Initially, lesions are superficial and seen as discolored areas and slight hyperkeratosis of the footpad and hock skin [6,7]. These lesions might progress into deep ulcers and necrosis of the epidermis, replaced by necrotic and suppurative material [7,8]. In severe cases, breast ulcers may also develop covered by necrotic tissue and subcutaneous edema [7]. FPD may serve as a port of entry for microorganisms present in the environment, leading to a systemic infection [9]. FPD and HB can be induced by wet litter and high ammonia concentrations [10,11]. However, the mechanism for the development of HB can differ from FPD lesions, as HB is more related to weight and less to litter characteristics [12]. Moreover, different litter materials significantly influence both the foot health of the chickens and the microbial load of the litter [13].

Dirty feathers is a welfare indicator associated with the condition of the litter, which may be wet, soiled, or contaminated with feces. Assessments of dirty feathers can be conducted on farms or in the slaughterhouse and provide valuable insights into the living conditions of the birds [5]. Birds that are dead on arrival (DoA) and condemnation of carcasses due to conditions such as emaciation, ascites, cellulitis, respiratory problems, and leg and wing fractures are appreciable causes of loss for the broiler industry and a reflection of poor bird health and welfare on farms or during transport [14,15,16]. Longer transport and lairage durations are also associated with an increase in the DoA rate [16]. Minimizing mortality in a flock is crucial not only for profit but also for maintaining a high standard of health and welfare. Furthermore, particular healthcare costs increase as the assumed level of animal welfare increases; this does not apply to all diseases and is most likely to be outweighed by other costs [17]. Emaciation is defined as wasting due to malnutrition, starvation, or inability to reach food [18], and the carcasses are identified on the slaughter line as being smaller due to the wasting of the pectoral muscles [18,19]. Ascites is a serious condition in which fluid accumulates in the abdominal cavity [20]. This can cause progressive and distressing symptoms, such as breathing difficulties [21]. This condition occurs when broilers are unable to provide enough oxygen to meet their metabolic needs, and it often arises in low-temperature environments and when the birds are fed with high-energy content feed [22]. In cold weather, when carried out in order to maintain the house temperature, s reduction in ventilation has been found to be a risk factor for the increased incidence of ascites [23].

Flock “thinning” is a common practice, especially in broiler flocks, and involves removing a portion of birds from the flock based on their weight to meet market demand for both lighter and heavier birds [15]. This approach allows for operating with more birds with stocking densities of 33 kg/m^2^ [1,5]. However, handling the birds during “thinning” may lead to flock stress and extra movement of the birds, increasing the risk of skin scratches and hemorrhages, which may develop into dermatitis and cellulitis and compromise biosecurity [14]. Lighting and litter quality are important aspects of poultry management on farms that may affect the incidence of scratches and cellulitis [24]. Moreover, different lighting programs and intensities may have implications for the behavior and physical activity of broilers and can be used to improve their welfare [24].

To the best of our knowledge, there are areas for improvement on farms regarding the main factors responsible for poor welfare conditions observed during the assessments performed at slaughterhouses in accordance with the Broiler Directive (Council Directive 2007/43/EC). A common criterion used in animal welfare assessment is a multi-criteria approach based on the assumption that suffering from several lesions is generally worse than suffering from a single lesion [25,26].

The objective of the present study was to assess the welfare of broilers at the flock level in three commercial slaughterhouses. This study evaluated broiler chickens of varying ages and weights using animal-based indicators. Additionally, integrated farms that received unsatisfactory welfare results at these slaughterhouses were audited.

It has been hypothesized that animal-based indicators collected in slaughterhouses could serve as indicators of the underlying production management systems. Additionally, they may help to identify specific areas within those systems that require improvement.

## 2. Materials and Methods

The first component of this study was performed in poultry slaughterhouses in Portugal between October 2019 and March 2020 (6 months), which are the coldest and wettest months, with average temperatures between −2 °C (minimum) and 24 °C (maximum). The welfare of 70 different batches of transport from distinct farms, with a total of 327,066 birds, was evaluated. These flocks were assessed in three slaughterhouses (A, B, and C) from three different companies: 21 flocks were in slaughterhouse A, 30 flocks were in slaughterhouse B, and 19 flocks were in slaughterhouse C. Broilers were a fast-growing genotype (Ross 308). Slaughterhouses A and C were considered to have small dimensions, slaughtering 2,687,598 and 1,970,131 birds in 2020, respectively. Slaughterhouse B is larger and slaughtered 9,096,024 birds in 2020. Flocks were, on average, 33 days (±5.7) old, ranging between 25 and 45 days, with an average body weight (BW) of 1.51 kg (±0.41). The average ages (days) and BWs (kg) of birds in slaughterhouses A, B, and C were 30.9 (±3.6) days and 1.47 (±0.28) kg, 37.3 (±5.3) days and 1.73 (±0.49) kg, and 29.1 (±3.2) days and 1.28 (±0.15) kg, respectively. For each flock, data were collected at the slaughter line at different points using the scoring scales mentioned below. A random sample of 100 broilers was applied for each measurement, including dirty feathers (DFs), footpad dermatitis (FPD), hock burns (HB), and breast lesions. FPD and HB were observed on the right foot and the right hock per carcass, as described by Ekstrand et al. [27]. The classification of the measures collected in the slaughterhouses was completed according to the following description, which was applied by Saraiva et al. [5]:

DF: 0 = clean feathers (white feathers with the absence of dirt), 1 = moderately dirty feathers (soiling feathers localized in the breast and abdominal areas without caked dirt), and 2 = very dirty feathers (generalized dirty brown feathers sometimes with dirt adhered or caked to feathers) (adapted from [25]);

FPD: 0 = no lesions (no visible lesions: smooth epidermis, no discoloration), 1 = mild lesions (papillae only with hyperkeratosis and/or mild/superficial lesions with discoloration or erosions in the epidermal layer up to 5 mm) and 2 = severe lesions (severe papillae and ulcerations: discoloration, hyperkeratosis, ulcers, and signs of inflammatory reactions with more than 5 mm) [27,28];

HB: 0 = no lesions (no visible lesions), 1 = mild lesions (brown lesion up to 5 mm), and 2 = severe lesions (black lesion with more than 5 mm) (adapted from [26]);

BB: 0 = no lesions (no visible lesions) and 1 = one or more breast burns with a brownish-colored scab (erosion) [29];

Breast ulcer: 0 = absence and 1 = breast skin covered with black exudates [29];

Breast blister: 0 = absence and 1 = breast blister equal to or larger than 0.5 cm^2^ [26].

Concerning the causes of total condemnation, a random selection of 200 carcasses and respective offal were observed per batch for emaciation, septicemia/abnormal color, cellulitis, ascites, and extensive traumatism. Total condemnations not related to the health and welfare of birds, including mechanical trauma and fecal contamination, were not included in this study. Information concerning the identification of farms, the age of birds, BW, DoA birds, total slaughtered birds, and *post-mortem* condemnations were collected from the slaughterhouse records. The thresholds for FPD, HB, and DFs were categorized into two grades, grade 1, or moderate (warning), and grade 2, or severe (alarm). The presence of BB, breast blisters, and breast ulcers was evaluated using a single threshold grade, as previously mentioned. The thresholds for extensive injuries above 2%, condemnation rates above 4%, and DoA rates above 0.5% were also applied in accordance with the guidelines set by the competent authority of Portugal for evaluating animal welfare indicators at slaughterhouses [30].

To investigate this further, the second component of this study was conducted on poultry farms. In case of threshold achievement in the same flock, two or more of the conditions mentioned above the farms were audited. On-farm factors such as feeder and drinker space, litter material, litter quality and integrity, stocking density, bird health, daily mortality, cumulative daily mortality, the presence of FPD lesions, panting, and lameness were monitored [2]. Under Council Directive 2007/43/EC, all chickens must have permanent access to litter that is dry and friable on the surface [1]. The evaluation of litter integrity and quality was carried out by checking for points of wet litter and difficulty moving leaving foot imprints or causing litter to stick to boots. During the inspections, the temperature, humidity, lighting program, light intensity, and ammonia level were verified using a thermo-hygrometer (KlimaLogg Pro), a luxmeter PCE-170 (PCE Instruments), and Detector Dräger-Tubes for Ammonia measurements (Standard Measuring Range: 5 to 70 ppm) using “Dräger-Tube pump accuro”. The light intensity was assessed at the birds’ eye level and at 10 distinct points within each house. The mean value of these measurements was subsequently presented. For audit control, seven farms were selected. However, during the scheduled visits, it was found that two of the farms had been closed down. Therefore, only 5 farms with a total of 11 houses were visited. The visits were scheduled one to five days before the first “thinning” for slaughter. This study was conducted under the supervision of the official authorities.

### Statistical Analysis

One-way ANOVA was performed to determine the significance of differences in mean age and BW between slaughterhouses, followed by Tukey’s HSD test for pairwise comparisons. The associations (*p* < 0.05) between the slaughterhouses (A, B, and C) and eighteen variables (DF0, DF1, and DF2; FPD0, FPD1, and FPD2; HB0, HB1, and HB2; BB0 and BB1; emaciation; septicemia/abnormal color; cellulitis; ascites; extensive traumatism; total condemnation; and DoA rate) were studied using nonparametric tests (Kruskal–Wallis test). To explore the relationships between variables, principal component analysis (PCA) was applied. In the first approach, all the variables were used to determine the first three principal components (PC). A reduction in the number of variables was carried out since some did not have a significant impact on the outcome. Eight variables were chosen based on factor loading (FL) moduli higher than 0.50 in absolute values and communalities (CM) higher than 0.5. The variables selected were emaciation, ascites, FPD1, FPD2, HB0, HB2, BB0, and BB1. Appropriateness was confirmed by Bartlett’s sphericity test (*p* < 0.0001). The number of components retained in the final solution was based on the Kaiser–Meyer–Olkin (KMO) criterion for the analysis of eigenvalues (>1) and the proportion of variance retained (>80%). Data analysis was carried out using the statistical software IBM SPSS Statistics for Windows (Version 26.0).

## 3. Results

In Table 1, the mean values (±SD) of age (days) and BW (kg) for each slaughterhouse (A, B and C) are presented.

Slaughterhouse B presented statistical differences to slaughterhouses A (*p* < 0.001) and C (*p* < 0.001) regarding the ages of birds at slaughter. Concerning the BWs of birds, the three slaughterhouses differed statistically (A vs. B, *p* < 0.001; A vs. C, *p* = 0.006; B vs. C, *p* < 0.001).

Welfare results obtained for each measure were registered per flock in each slaughterhouse (Table 2). The average condemnation and DoA rates per flock were 1.40% (range between 0.10% and 10.48%) and 0.30% (range between 0% and 2.83%), respectively. On average, emaciation was the first cause of condemnation (0.60%), followed by septicemia/abnormal color (0.49%), cellulitis (0.15%), and ascites (0.10%). Extensive trauma was present in 0.01% of the slaughtered animals, though no flock reached the threshold of 2%.

Skin lesions (FPD, HB, and BB) were more frequent in slaughterhouse C, and significant differences were observed for all grades of HB and FPD between slaughterhouses A and C. In the present study, from the 70 batches assessed, only three flocks had condemnation rates higher than 4%, and eleven flocks had DoA rates higher than 0.5%. Twenty-one flocks achieved a warning for FPD and fourteen achieved an alarm for FPD, resulting in 50% of flocks having FPD lesions. Regarding HB, one flock was rated as grade 2 and two flocks scored grade 1. However, these three flocks presented grade 2 for FPD. In slaughterhouses A and B, emaciation was the major cause of condemnation, accounting for 0.59% and 0.71% of rejections, respectively. In slaughterhouse C, abnormal color was one of the major causes of condemnation (0.49%), followed by emaciation (0.45%). Ascites and cellulitis were fairly important causes of condemnation; however, they were less prevalent than emaciation and abnormal color. In slaughterhouse B, cellulitis was the third cause of condemnation (0.30%) and the fourth cause in slaughterhouses A and C, with mean values of 0.06% and 0.03%, respectively. Slaughterhouses A and C showed significant differences from slaughterhouse B regarding the condemnation rates related to ascites (*p* = 0.002) and cellulitis (*p* = 0.003), with slaughterhouse B showing the highest percentages.

Factor loadings for emaciation, FPD1, FPD2, HB0, HB2, BB0, and BB1 after varimax normalized rotation, and the communalities from three PCs are presented in Table 3.

The factor loadings for FPD1 were −0.10 for PC1, 0.03 for PC2, and 0.98 for PC3. Additionally, FPD1 presented a communality of 0.98, suggesting that FPD1 is an important contributor to the overall variance in the analysis. The factor loadings for HB2 were 0.96 for PC1, 0.28 for PC2, and -0.14 for PC3. HB2 was strongly associated with BB1 and moderately associated with FPD2 in PC1, as evidenced by its high factor loading. The substantial communality value of 0.92 indicates that HB2 significantly contributes to the overall variance in the analysis, underlining its importance in the dataset. PC1 accounts for a significant portion of the overall variance, with FPD2, HB0, HB2, BB0, BB1, and emaciation presenting high factor loadings in absolute terms, indicating their essential roles as the most influential variables in the analysis.

**Figure 1:**
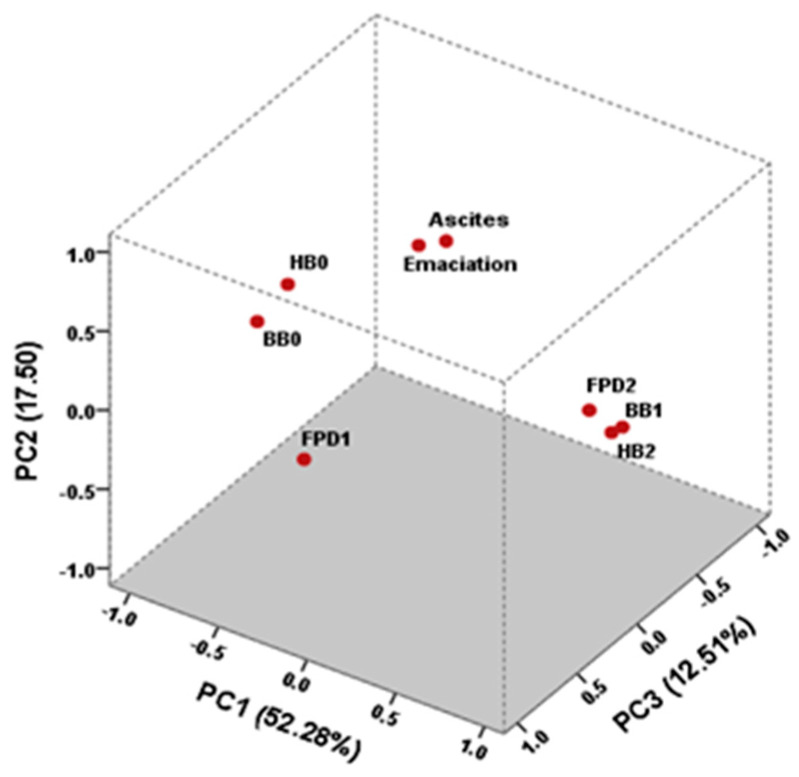
Loadings after varimax normalized rotation of the eight variables: emaciation, ascites, mild footpad dermatitis (FPD1), severe footpad dermatitis (FPD2), the absence of hock burns (HB0), severe hock burns (HB2), the absence of breast burns (BB0), and the presence of breast burns (BB1) to three-dimension principal component analysis (PCA).

Figure 1 shows the projection of the eight variables in the three-dimensional space. PC1 explains 52.28% of the total variance, PC2 explains 17.50%, and PC3 explains 12.51%. The PC1 was mainly defined by FPD2, HB2, and BB1, and their relationship is shown on the right side of the figure. BB1 presents the highest loading with 0.97, followed by HB2 with 0.96. In this case, while FPD2 does not have the highest loading, its relationships with HB2 and BB1 may have made it an important contributor to the PC1. Also, BB0 and HB0 are highly associated on the left side, with both factorial loadings being higher than 0.92 (in absolute value), indicating a strong relationship between these variables. The most important contributions to the PC2 came from emaciation and ascites, which were greatly correlated with factorial loadings higher than 0.82.

Table 4 displays the alarm/warning thresholds observed at slaughterhouses, accompanied by data collected during welfare farm audits. Farms I and II were part of a vertically integrated system with slaughterhouse B, while farms III and V were affiliated with slaughterhouse A. Farm IV operated under vertical integration with slaughterhouse C. These relationships highlight the integrated management of farms and slaughterhouses within the same company or cooperative structure. The data from tables include variables such as age, body weight (BW), bird density, ventilation, heating, feeder, drinker space, and the type of drinker used in various houses.

The table above provides an overview of various parameters related to different houses, offering insights into their management practices and conditions. Farms utilize different ventilation systems, with varying numbers of fans and window configurations. Farms I and II have more fans compared to farms III, IV, and V. Heating setups differ across farms, with varying numbers of heaters distributed throughout the houses. The arrangement of feeders and drinkers was similar across farms, with 3 to 4 rows of drinkers interspersed with feeders. However, the specific configuration may vary slightly. Farms use different types of drinkers, including automatic nipples and bell drinkers.

The birds on farm I were between 23 and 31 days old, and the average live weight was between 0.651 kg and 1.832 kg. At the moment of the visit, farm I presented densities within the stipulated limits (<33 kg/m^2^). However, this means, for instance, in house 1 when birds reach 1.20 Kg of BW, that the stocking density will be 33 kg/m^2,^ and at this time, the birds do not have enough BW for the first “thinning”. On different farms, birds of the same age (27 days) had different BWs, as observed for farms I, II, III and IV, with 0.901 kg, 1.761 kg, 1.111 kg, and 1.170 kg, respectively. Stocking density refers to the total live weights of broilers in a house per square meter of usable area, expressed in kilograms per square meter (kg/m^2^). At 27 days, stocking densities varied across farms, with farm III presenting 21.44 kg/m^2^ and farm IV reaching a stocking density of 29.34 kg/m^2^. Farm IV presented the highest stocking densities among all houses, nearing the 33 kg/m^2^ limit despite not having the heaviest average birds.

Table 5 indicates the results from different parameters evaluated in the 11 houses one to five days before the first “thinning” for slaughter.

Only on farm I was it possible to verify the CO_2_ concentration, as it was monitored by the computer system used, and regarding ammonia, no house exceeded the legal limit of 20 ppm for the concentration of this gas. On farm I, the litter was in poor condition in both houses, with areas of high humidity or moisture. Additionally, there was an issue with the pipette system, which appeared to be leaking water, exacerbating the problem near the drinking fountains. Moreover, flocks from house 1 did not have an adequate period of darkness: 24 h light is only legal in the three days before slaughter, which was not the case because the animals were slaughtered five days after this control. In house 2, although there was a period of darkness, it was divided into four periods of 1 h and 30 min, which does not meet the established in Council Directive 2007/43/EC determining that at least 4 h of darkness should be uninterrupted. The ammoniacal odor was notorious in house 2, even though the ammonia concentration measure (12 ppm) was within the legal limits. Farm II applied a light program appropriate for the needs of the animals, though the intensity of light in house 2 was decreased. On farms III, IV, and V, birds did not meet the requirements for the light program, as the broilers were not subjected to an uninterrupted dark period of 4 h, totaling 6 h of dark in a 24 h cycle.

## 4. Discussion

The mean BWs of broilers at slaughter differed statistically between slaughterhouses, with slaughterhouse B showing a higher BW of 1.73 (±0.49) kg, followed by slaughterhouse A with 1.47 (±0.28) kg and C with 1.28 (±0.15) kg. Slaughterhouses A and C slaughter “barbecue chicken”, while slaughterhouse B slaughters “barbecue chicken” and heavier broilers. Broilers with more FPD lesions (FPD1 = 46% and FPD2 = 39.2%) were observed in slaughterhouse C, and better scores for this indicator were observed in slaughterhouses A (FPD1 = 23.7% and FPD2 = 13.05%) and B (FPD1 = 32% and FPD2 = 15.4%). Statistical differences between flocks from slaughterhouses A and C were observed for both FPD grades. The data suggest that the company operating slaughterhouse C typically maintains more birds per square meter compared to those associated with the other two slaughterhouses. This could be attributed to the fact that they process younger birds with lower body weights (averaging 29 days and 1.28 ± 0.15 kg). It seems that a combination of factors, such as high stocking densities and probably poor environmental conditions such as deficit of ventilation and heating, may have contributed to the pad lesions observed in slaughterhouse C flocks. These conditions were verified during audits on farm IV, which is vertically integrated with the same company that owns slaughterhouse C and, for that reason, slaughters its birds in this slaughterhouse. On the other hand, slaughterhouse A achieved better welfare outcomes in FPD, HB, abnormal color/septicemia, and extensive trauma compared to other slaughterhouses (Table 2). During the audit of farm III, which presented unsatisfactory results at slaughterhouse A for FPD and DoA, it was observed that the stocking density was in compliance with Council Directive 2007/43/EC [1].

In PCA analyses, the association in PC1 between HB0 (FL = −0.92) and BB0 (FL = −0.97) and in PC3 between FPD1 (FL = 0.98) and BB0 (FL = 0.94) corroborate the idea that lesions on the tibiotarsal skin surface and breast develop late, followed by those affecting the pads. Saraiva et al. [5] studied the incidence of FPD and HB and the relationship between them and also observed that footpad lesions tend to develop before hock burns, indicating a sequential progression in the development of these lesions caused by prolonged contact with poor litter quality [31]. The positive correlation between FPD2, HB2, and BB1 suggests that these conditions tend to occur simultaneously (Figure 1). Similar data were reported by Allain et al. [26], who observed a positive correlation between percentages of BB and black HB (r = 0.45, *p* < 0.01). In the present study, the results obtained for HB1 and HB2 differ in slaughterhouses A and C, with slaughterhouse C again showing the worst results (HB1 = 16.95% and HB2 = 4.79%). Nevertheless, no differences in HB1 and HB2 values are observed between slaughterhouses A or C and B (HB1 = 4.57% and HB2 = 1.37%), with the latter presenting the heaviest chickens. Consequently, we assume that higher percentages of HB1 and HB2 are observed in lighter chickens than expected. For instance, other experiments found a positive correlation between HB and BW, showing that the heavier birds were more affected by HB [8,32]. In a study conducted in a broiler slaughterhouse of similar size to slaughterhouse B, there was an observation of 7.34% HB1 and 2.33% HB2 lesions, totaling 9.67% of HB lesions [5]. According to Hepworth et al. [33], the presence of HB is an indicator of broiler flock health, and their results showed that it was associated with the rearing system, growth rate, days from first “thinning”, transit mortality, and carcass downgrades. Moreover, through the investigation of warning indicators for HB in broiler flocks, their findings indicated that birds were influenced by specific factors of that particular house and that birds differed more from one another if reared in a different house [34]. Factors of particular houses, such as high levels of humidity and difficulties in maintaining adequate temperature and ventilation in old farms, which are essential to reduce ammonia concentration [26], could explain the worse scores of FPD and HB in slaughterhouse C, even though the birds were slaughtered at an average of 29 days. Increased water consumption may reduce the quality of the litter both directly, through increased excretion, and indirectly, through spillage [34]. Additionally, nutrition is an important factor affecting water intake, excreta moisture, and litter quality, thereby influencing the occurrence and intensity of FPD in birds [35].

According to Shynkaruk et al. [36], decreased stocking density leads to reduced litter moisture and a decline in footpad dermatitis. Furthermore, lower stocking densities corresponded with higher final body weights, and these environments exhibited lower rates of infectious mortality. It was observed that farm I had a higher stocking density and also presented more warnings/alarms for four welfare indicators: FPD, HB, DoA, and condemnation rate (Table 4). This again suggests a potential correlation between the increase in stocking density and the poor welfare indicators observed on the farm. This result is in line with Dawkins et al. [28] and Bokkers et al. [37], showing that higher densities are associated with reduced welfare parameters such as litter quality and the behavioral opportunities of broiler birds. For Sørensen and Fraser [32], higher stocking densities are associated with more FPD and HB, and these results can reflect poorer litter quality, indicated by the increased moisture content of the litter from pens housed at higher densities. Moreover, in agreement with Dozier et al. [38], high densities dramatically reduce the growth rates of birds and the quality of carcasses, increasing the prevalence of FPD by reducing the litter quality. The average condemnation rate per flock of 1.40 (range between 0.10% and 10.48%) was similar to those obtained by Haslam et al. [14], Saraiva et al. [5], and Junghans et al. [39], with an average rejection rates of 1.23 (range 0.07 and 5.51%), 1.53 (ranging from 0.32 to 7.29%), and 1.48 (ranging from 0.12 to 4.63%), respectively. In the present study, the most frequent cause of condemnation was emaciation (0.60%), followed by abnormal color/septicemia (0.49%), cellulitis (0.15%), and ascites (0.10%). The percentage of emaciated birds at the slaughterhouse reflected this condition on farms and represented the most frequent cause of condemnation in slaughterhouses A and B and the second most frequent cause in slaughterhouse C. Emaciation has high loadings on PC1 (FL = −0.79) and PC2 (FL = 0.82), indicating that it is strongly associated with these two principal components. However, differences in the condemnation rates of small broilers could have been due to differences in disease occurrence on farms or culling management within companies [40]. Moreover, PCA indicated the simultaneous occurrence of emaciation (FL = 0.82) and ascites (FL = 0.84). The relatively high factor loadings, particularly above 0.8, on the same principal component indicate a simultaneous occurrence or significant relationship between emaciation and ascites. This means that when one of these conditions is present, the likelihood of the other being present is also high [5]. An increased prevalence of these broilers is a sign of the weakened welfare of the on-farm broiler flock [20,38] and is usually a sign of long-standing issues such as locomotor problems and an inability to reach food [41]. Slaughterhouse A (0.13 ± 0.02%) and C (0.06 ± 0.13%) showed significant differences from slaughterhouse B regarding the condemnation rates by ascites (*p* = 0.002), with slaughterhouse B showing the highest percentages (0.20 ±0.27%). The prevalence of ascites was notably higher in slaughterhouses with higher slaughter ages (37.3 versus 30.9 and 29.1), a trend consistent with the findings of Forseth et al. [42]. Their study revealed a significant increase in ascites’ prevalence with higher slaughter age. Ascites arise as a consequence of pulmonary hypertension syndrome, often attributed to rapid growth rates, and it is a significant condemnation cause in many European countries such as Denmark (0.20%) [15], the UK (0.45%) [43], and Germany (0.53%) [39]. Furthermore, when assessing the prevalence of condemnation causes in broilers, it is critical to consider the slaughter age alongside other predisposing factors. The low temperatures in cold weather may force farmers to reduce ventilation to keep the poultry houses warm, leading to high levels of ammonia, which predisposes lung diseases and ascites [21], and this might be the main circumstance responsible for the development of ascites in broilers from the present study, which was performed throughout cold weather. Overall, the findings from our study suggest that enhancing environmental and management conditions on visited farms, such as heating, ventilation, and light programs, while also reducing stocking densities could significantly improve animal welfare to meet acceptable standards. This aligns with the research conducted by Dawkins et al. [28] and Xavier et al. [44], which emphasizes the crucial role of the overall farm environment in determining welfare outcomes beyond stocking density. After collecting animal-based indicators from slaughterhouses, it was observed that significant welfare concerns persist in some farms in the production of broiler chickens in Portugal, despite the implementation of Council Directive 2007/43/EC. High scores of FPD and HB were observed in some flocks. Condemnation rates exceeding 4% were observed in three flocks, and 11 flocks presented DoA rates higher than 0.5%. These findings indicate ongoing challenges involved in ensuring optimal welfare conditions for broilers. Council Directive 2007/43/EC served as the basis for conducting thorough audits of farms to ensure adherence to welfare standards. These audits must be conducted transparently and subjected to independent scrutiny to maintain accountability and ensure the integrity of the auditing process.

## 5. Conclusions

This study has provided information regarding the causes of condemnations in Portugal and the principal welfare indicators that can be used as baseline data for the implementation of actions at the farm level. The high prevalences of severe footpad dermatitis (FPD2), severe hock burns (HB2), and condemnations observed at the slaughterhouses were perceived as relevant indicators, determining visits to the farms for welfare assessments. Litter humidity was observed in all audited farms, which may be influenced by environmental stressors like poor ventilation and high ammonia levels, therefore having a detrimental impact on welfare. Audits on farms are crucial for indicating the need for improvement in environmental conditions such as house ventilation, temperature, and light programs, as well as decreasing stocking densities. This study highlights the need to continue monitoring and improving the welfare of broiler chickens in the EU, even after the implementation of EU legislation. By conducting studies that investigate the welfare of broiler chickens using animal-based indicators and auditing production management systems, we can identify areas for improvement and work towards ensuring that broiler chickens are kept in conditions that promote their health and welfare.

## Figures and Tables

**Table 1:** Mean values (±SD) of age (days) and BW (kg) in each slaughterhouse (A, B, and C).

Variables	Slaughterhouse A (n = 21)	Slaughterhouse B (n = 30)	Slaughterhouse C (n = 19)
Age (days)	30.9 ± 3.6 ^a^	37.3 ± 5.3 ^b^	29.1 ± 3.2 ^a^
BW (kg)	1.47 ± 0.28 ^a^	1.73 ± 0.49 ^b^	1.28 ± 0.15 ^c^

^a–c^ Means within a row with different superscripts differ (*p* < 0.05). n = number of flocks. BW = body weight.

**Table 2:** Mean percentages (±SD) of welfare indicators results per slaughterhouse (A, B, and C).

Measures (%)	Slaughterhouse A (n = 21)	Slaughterhouse B (n = 30)	Slaughterhouse C (n = 19)	*p*-Value
DF0	67.19 ± 13.62	68.23 ± 14.56	70.37 ± 21.47	NS
DF1	26.76 ± 11.13	25.73 ± 12.18	23.58 ± 16.46	NS
DF2	6.00 ± 3.65	6.03 ± 4.81	6.58 ± 5.91	NS
FPD0	63.67 ± 19.18 ^a^	52.27 ± 22.46 ^a^	14.79 ± 24.43 _b_	0.000
FPD1	23.67 ± 15.01 ^a^	32.00 ± 15.12 ^ab^	46.0 ± 30.90 ^b^	0.038
FPD2	13.05 ± 9.63 ^a^	15.40 ± 15.23 ^a^	39.21 ± 36.80 ^b^	0.038
HB0	96.95 ± 4.87 ^a^	94.07 ± 10.14 ^ab^	78.37 ± 28.57 ^b^	0.042
HB1	3.05 ± 4.87 ^a^	4.57 ± 6.89 ^ab^	16.95 ± 19.61 ^b^	0.037
HB2	0.00 ± 0.00 ^a^	1.37 ± 4.06 ^ab^	4.79 ± 11.52 ^b^	0.026
BB0	99.86 ± 0.66	98.47 ± 3.82	96.53 ± 8.41	NS
BB1	0.14 ± 0.66	1.53 ± 4.17	3.47 ± 8.37	NS
Emaciation	0.59 ± 1.29	0.71 ± 0.62	0.45 ± 0.99	NS
Abnormal color/septicemia	0.32 ± 0.46	0.61 ± 0.37	0.49 ± 0.88	NS
Ascites	0.13 ± 0.02 ^a^	0.20 ± 0.27 ^b^	0.06 ± 0.13 ^a^	0.002
Cellulitis	0.06 ± 0.09 ^a^	0.30 ± 0.44 ^b^	0.03 ± 0.05 ^a^	0.003
Extensive trauma	0.00 ± 0.00 ^a^	0.00 ± 0.01 ^a^	0.02 ± 0.01 ^b^	0.000
Total condemnation	1.10 ± 1.79	1.80 ± 0.91	1.06 ± 2.30	NS
DoA	0.29 ± 0.44	0.41 ± 0.60	0.13 ± 0.35	NS

^a,b^ In each row, means with different letters differ significantly (*p* < 0.05); NS = not significant (*p* ≥ 0.05). n = number of flocks.

**Table 3:** Factor loadings and communalities of the first three PCs for emaciation, ascites, mild footpad dermatitis (FPD1), severe footpad dermatitis (FPD2), the absence of hock burns (HB0), severe hock burns (HB2), the absence of breast burns (BB0), and the presence of breast burns (BB1) after varimax normalized rotation.

Variable	Factor Loadings			^b^ CM
	^a^ PC1	PC2	PC3	
Bartlett’s test of sphericity	< 0.001			
^c^ KMO measure	0.66			
Mild footpad dermatitis (FPD1)	−0.10	0.03	**0.98**	0.98
Severe footpad dermatitis (FPD2)	**0.69**	**−0.78**	−0.23	0.53
Absence of hock burns (HB0)	**−0.92**	0.12	−0.10	0.88
Severe hock burns (HB2)	**0.96**	0.28	−0.14	0.92
Absence of breast burns (BB0)	**−0.97**	−0.14	**0.94**	0.94
Breast burns (BB1)	**0.97**	0.14	**−0.84**	0.94
Emaciation	**−0.79**	**0.82**	**0.60**	0.68
Ascites	0.23	**0.84**	−0.19	0.71
Eigenvalues	4.18	1.40	1.00	
Explained variance (%)	52.28	17.50	12.51	Σ = 82.29

^a^ PC—principal component; ^b^ CM—communality; ^c^ KMO—Kaiser–Meyer–Olkin.

**Table 4:** Slaughterhouse thresholds and data from different houses (area, age, BW, density, ventilation, heating, feeder and drinker space, drinker type, and litter condition).

Farm	Slaughterhouse (Thresholds)	House (Area in m^2^)	Age (Day)	BW (kg)	Stocking Density (kg/m^2^)	Ventilation	Heating	Feeders and Drinker Space	Type of Drinker	Litter
I	B (FPD, HB, DoA, and Condemnation rate)	1 (1452)	27	0.901	24.75	16 automatic fans (48 air outs on the opposite side)	8 heaters distributed	4 rows of drinkers interspersed with 3 rows of feeders	Automatic nipples	Several areas of humidity, nipple system with water spillage
		2 (1452)	23	0.651	17.61					
II	B (FPD and Condemnation rate)	1 (1100)	31	1.807	29.82	6 fans (48 manual opening windows)	7 heaters distributed	4 rows of drinkers interspersed with 3 rows of feeders	Bell drinkers	Areas of humidity
		2 (1100)	31	1.832	29.63				Bell drinkers	
		3 (1100)	27	1.761	29.38				Automatic nipples	
III	A (FPD, DoA)	1 (765)	27	1.111	21.44	1 fan (45 manual opening windows)	1 heater	3 rows of drinkers interspersed with 2 rows of feeders	Automatic nipples	Areas of humidity
IV	C (FPD, HB)	1 (560)	27	1.170	29.34	2 fans (56 manual opening windows)	2 heaters	4 rows of drinkers interspersed with 3 rows of feeders	Bell drinkers	Some humidity next to the drinkers
V	A (FPD, HB, and Condemnation rate)	1 (1171)	28	1.040	17.89	3 fans (112 manual opening windows)	2 heaters	4 rows of drinkers interspersed with 3 rows of feeders	Automatic nipples	Humidity points on the ceiling and infiltrations into the floor
		2 (1171)	28	1.020	20.36					
		3 (1171)	29	1.150	21.56					
		4 (1171)	29	0.997	20.76					

FPD—footpad dermatitis; HB—hock burns; DoA—dead on arrival.

**Table 5:** Temperature, moisture, NH_3_ and CO_2_ levels; light intensity measured during the audit; and the light/dark regime applied in each house.

Farm	House	Temperature (°C)	Moisture (%)	NH_3_ (ppm)	CO_2_ (ppm)	Light Measured	Light Intensity (lux)	Light/Dark Period
I	1	25.1	63	5	1748	artificial	62.9	24 h light/0 h dark
	2	25.4	62	12	1900	artificial	74.3	(4.5 h light/1.5 h dark) × 4
II	1	24.8	58	5	-	artificial	75.9	13 h light/5 h dark and 4 h light/2 h dark
	2	25.2	61	8	-	natural	7.8	
	3	23.3	69	15	-	artificial	12.6	
III	1	24.6	61	18	-	natural	80.3	22 h light/2 h dark
IV	1	20.0	59	3	-	natural	6.6	22 h light/2 h dark
	-	-	-	-	-	artificial	36.0	
V	1	24.7	-	10	-	artificial	30.4	20 h light/4 h dark
	2	24.6	-	12	-	artificial	15.0	
	3	24.5	-	5	-	artificial	17.5	
	4	28.0	-	5	-	artificial	18.7	

## Data Availability

The authors do not have permission to share data.
